# Revealing unconventional host–guest complexation at nanostructured interface by surface-enhanced Raman spectroscopy

**DOI:** 10.1038/s41377-021-00526-5

**Published:** 2021-04-19

**Authors:** Gan-Yu Chen, Yi-Bin Sun, Pei-Chen Shi, Tao Liu, Zhi-Hao Li, Si-Heng Luo, Xin-Chang Wang, Xiao-Yu Cao, Bin Ren, Guo-Kun Liu, Liu-Lin Yang, Zhong-Qun Tian

**Affiliations:** 1 State Key Laboratory of Physical Chemistry of Solid Surfaces, Collaborative Innovation Center of Chemistry for Energy Materials (iChEM), College of Chemistry and Chemical Engineering, Xiamen University, Xiamen, 361005 China; 2 State Key Laboratory of Marine Environmental Science, Fujian Provincial Key Laboratory for Coastal Ecology and Environmental Studies, Center for Marine Environmental Chemistry & Toxicology, College of the Environment and Ecology, Xiamen University, Xiamen, 361102 China; 3 School of Electronic Science and Engineering (National Model Microelectronics College), Xiamen University, Xiamen, 361005 China; 4 Key Laboratory of Chemical Biology of Fujian Province, Xiamen University, Xiamen, 361005 China

**Keywords:** Raman spectroscopy, Imaging and sensing

## Abstract

Interfacial host–guest complexation offers a versatile way to functionalize nanomaterials. However, the complicated interfacial environment and trace amounts of components present at the interface make the study of interfacial complexation very difficult. Herein, taking the advantages of near-single-molecule level sensitivity and molecular fingerprint of surface-enhanced Raman spectroscopy (SERS), we reveal that a cooperative effect between cucurbit[7]uril (CB[7]) and methyl viologen (MV^2+^2I^−^) in aggregating Au NPs originates from the cooperative adsorption of halide counter anions I^−^, MV^2+^, and CB[7] on Au NPs surface. Moreover, similar SERS peak shifts in the control experiments using CB[n]s but with smaller cavity sizes suggested the occurrence of the same guest complexations among CB[5], CB[6], and CB[7] with MV^2+^. Hence, an unconventional exclusive complexation model is proposed between CB[7] and MV^2+^ on the surface of Au NPs, distinct from the well-known 1:1 inclusion complexation model in aqueous solutions. In summary, new insights into the fundamental understanding of host–guest interactions at nanostructured interfaces were obtained by SERS, which might be useful for applications related to host–guest chemistry in engineered nanomaterials.

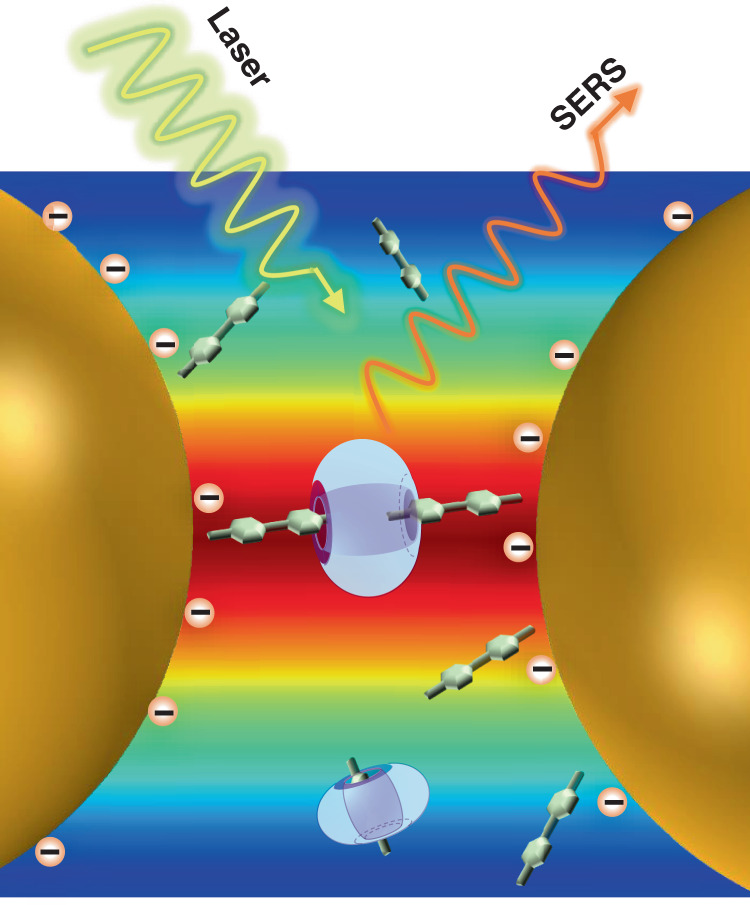

## Introduction

Host–guest chemistry offers a reversible and versatile way to achieve high-fidelity recognition between host and guest molecules; therefore, it is widely employed in homogeneous solutions^1–4^. In recent studies, host–guest systems have been used on solid surfaces to functionalize nanomaterials^5–12^. The results showed that the host–guest complexation behavior on surfaces may differ from those observed in solutions. For instance, host–guest complexation on surfaces may greatly enhance compared with solutions. This can be explained by the preorganization of ligands on surfaces, as well as the synergistic effects induced by multiple noncovalent interactions^5^. Besides, competitive adsorptions on surfaces may lead to unpredictable surface environments, thereby affecting the interfacial host–guest complexation. As a result, it is important to better understand the interfacial behavior of host–guest systems for their wider and more robust applications in nanomaterials. However, the complex surface effects and trace amounts of components present on the surface make the study of interfacial complexation more difficult^13^.

Several techniques such as extinction spectroscopy, fluorescence spectroscopy^14–16^, and cyclic voltammetry^17,18^ have been used to monitor the host–guest complexation at interfaces. However, these methods often require the use of chromophores or redox-active motifs and suffer from background interferences generated by nanoparticles (NPs). Nuclear magnetic resonance (NMR) spectroscopy provides information about ligand-shell morphology or ligand exchange on metal NPs^19–21^, but suffers from relatively high detection limit and background interference caused by residual agents in solutions. Alternatively, surface-enhanced Raman spectroscopy (SERS) provides rich molecular vibrational information along with many advantages in terms of ultrahigh sensitivity, label-free and in situ detection, distinction among ligand types, and exclusive detection of adsorbates present on the surface^22,23^. Furthermore, SERS peaks of host and/or guest could shift upon host–guest complexation^13,24–27^, beneficial for investigating host–guest complexation at the interface. Therefore, SERS is a practical and powerful in situ technique for revealing interfacial host–guest complexation phenomena.

**Fig. 1:**
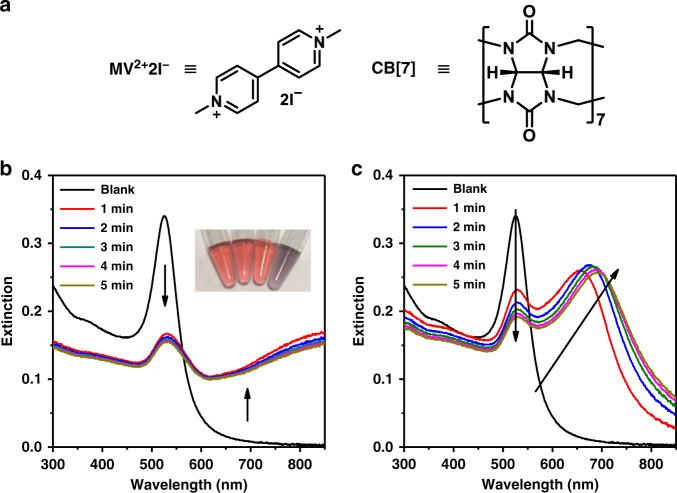
Host-guest solution induced aggregation of Au NPs. **a** Molecular structures of MV^2+^2I^−^ and CB[7]. **b** Time-dependent extinction spectra of Au NPs colloid obtained by adding MV^2+^2I^−^ and CB[7]. Inset shows the corresponding photographs of Au NPs colloid (from left to right) on the addition of H_2_O, MV^2+^2I^−^, CB[7], and MV^2+^2I^−^ + CB[7], respectively. The concentrations of MV^2+^2I^−^ and CB[7] are 2.5 μM and 0.31 μM, respectively. **c** Time-dependent extinction spectra of Au NPs colloid on the addition of 250 μM CB[7]

In this study, SERS was used to investigate host–guest interactions between cucurbit[7]uril (CB[7]) and methyl viologen (MV^2+^2I^−^) at Au NP-water interface (Fig. 1a). SERS studies showed that a cooperative effect between CB[7] and MV^2+^2I^−^ in regulating the aggregation of Au NPs originated from the cooperative adsorption of halide counter anions I^−^, MV^2+^, and CB[7] on Au NP surface. By carefully analyzing the SERS spectra at different molar ratios of host and guest as well as the Raman spectra in homogeneous solutions, we deduced that the complexation between MV^2+^ and CB[7] at the interface is different from that in solutions. Moreover, similar SERS peak shifts in the control experiments using CB[n]s but with different cavity sizes suggested the occurrence of same guest complexations among CB[5], CB[6], and CB[7]. Hence, we propose an unconventional exclusive complexation model between CB[7] and MV^2+^ on the surface of Au NPs which is distinct from the well-known 1:1 inclusion complexation model (Fig. S1) in aqueous solutions^28,29^.

## Results

### Cooperative effect of host and guest on Au NPs aggregation

In this study, a mixed solution of CB[7] (0.31 μM) and MV^2+^2I^−^ (2.5 μM) induced an instant aggregation of Au NPs (Fig. 1b). The time-dependent extinction spectra revealed a drastic decline in the localized surface plasmon resonance (LSPR) peak of Au NPs (ca. 526 nm) within 1 min (Fig. 1b), accompanied by the generation of a new LSPR peak above 800 nm. After 4 min, the spectrum remained almost unchanged, suggesting that the aggregation of Au NPs was almost completed within 1 min. In comparison, CB[7] or MV^2+^2I^−^ alone failed to produce the aggregation of colloidal Au at the same concentration within 5 min (Fig. S2). Though CB[7] alone at a higher concentration can also aggregate Au NPs^30,31^, the process was much less efficient (Fig. 1c). The much faster and efficient aggregation of Au NPs in the presence of CB[7] and MV^2+^2I^−^ suggested a cooperative effect of host and guest molecules in aggregating Au NPs.

**Fig. 2:**
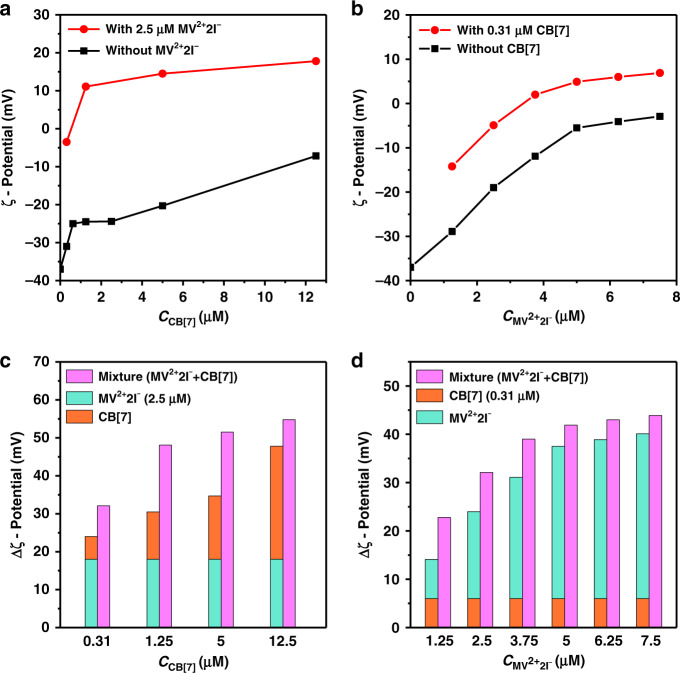
ζ-potential variations on the addition of host and/or guest solutions. **a** CB[7] concentration-dependent ζ-potential of Au NPs colloid with (red line) and without (black line) 2.5 μM MV^2+^2I^−^. **b** MV^2+^2I^−^ concentration-dependent ζ-potential of Au NPs colloid with (red line) and without (black line) 0.31 μM CB[7]. **c** and **d** Pink columns show the variations in ζ-potential (Δζ_mix_) with simultaneous additions of CB[7] and MV^2+^2I^−^. Orange and green columns display the sum in variations of ζ-potential (Δζ_sum_) caused solely by CB[7] and MV^2+^2I^−^

The cooperative effect of host and guest molecules in aggregating Au NPs was further confirmed from the variations in ζ-potential. To this end, two sets of ζ-potential measurements were carried out. The first ζ-potential was recorded as a function of the concentration of CB[7] with and without the addition of MV^2+^2I^−^. The second ζ-potential was recorded as a function of MV^2+^2I^−^ concentration with and without CB[7]. As shown in Fig. 2a, b, the addition of a mixture of CB[7] and MV^2+^2I^−^ led to a variation in ζ-potential more significantly than separately. To confirm the cooperative effect, two ζ-potential values were calculated and compared. The first is the change in ζ-potential (Δζ_mix_) on the addition of the mixture of CB[7] and MV^2+^2I^−^, and the second is the sum in the variations of ζ-potential (Δζ_sum_) caused solely by CB[7] and MV^2+^2I^−^. The data indicate that the value of Δζ_mix_ (Fig. 2c, d, pink column) at different molar ratios of host and guest molecules was ca. 9—58% higher than that of Δζ_sum_ (Fig. 2c, d, orange and green columns), confirming the cooperative effect of CB[7] and MV^2+^2I^−^ in regulating the aggregation of Au NPs.

### Cooperative adsorption of counter anions, MV^2+^, and CB[7] on surface

**Fig. 3:**
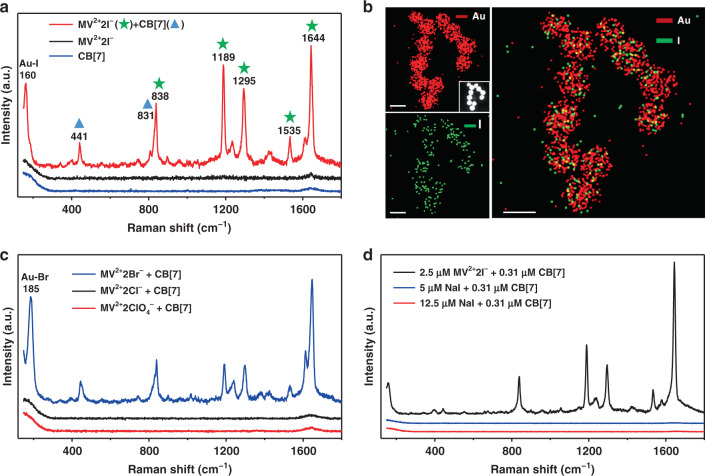
Cooperative adsorption of counter anions, MV^2+^, and CB[7] on Au NPs surface. **a** SERS spectra of Au NPs colloid on the addition of (MV^2+^2I^−^ + CB[7]), MV^2+^2I^−^, and CB[7], respectively. The concentrations of MV^2+^2I^−^ and CB[7] are 2.5 μM and 0.62 μM, respectively. **b** STEM elemental mappings of Au NPs aggregates on the addition of MV^2+^2I^−^ (2.5 μM) + CB[7] (0.31 μM). The inset shows the corresponding high-angle annular dark-field image. All the scale bars represent 50 nm. **c** SERS spectra of Au NPs colloid on the addition of MV^2+^2Br^−^ (2.5 μM) + CB[7] (0.31 μM) and MV^2+^2Cl^−^ (2.5 μM) + CB[7] (0.31 μM), respectively. **d** SERS spectra of Au NPs colloid on the addition of different concentrations of CB[7] + MV^2+^2I^−^/NaI

SERS studies at the molecular level revealed that the cooperative effect in aggregating Au NPs originated from the cooperative adsorption of halide counter anions, MV^2+^, and CB[7] on the surface of Au NPs. Strong SERS signals of both MV^2+^ and CB[7] were observed for the Au NPs colloid on the addition of a mixture of solutions of MV^2+^2I^−^ and CB[7] (Fig. 3a, red line). According to a previous study^32^ and the corresponding ordinary Raman spectra (Fig. S4), the SERS peaks at 838, 1189, 1295, and 1644 cm^−1^ mainly originated from C–C bond stretching, N-CH_3_ stretching, inter-ring C–C stretching, and ring C–C stretching vibration modes of MV^2+^. The two peaks at 441 and 831 cm^−1^ were assigned to the ring scissor and ring deformation modes of CB[n], respectively^33^. Only the in-plane modes of MV^2+^ and CB[7] were greatly enhanced, suggesting that they are located on the Au NPs surface with a perpendicular orientation according to the surface selection rules of SERS^34–36^. In addition, the SERS performance was found to be independent of the mixing order of CB[7], MV^2+^2I^−^, and Au NPs colloids, evidenced by the almost identical SERS performance in terms of peak locations and (relative) Raman intensities (Fig. S5). MV^2+^2I^−^ alone can also induce the aggregation of Au NPs and thereby produce a SERS signal (Fig. S6), but with 10 times concentration larger than those applied in host–guest induced aggregation (Fig. 3a and Fig. S5).

Interestingly, a strong Au-I band^37^ was observed at ca. 160 cm^−1^ for the Au NPs colloid on the addition of a mixture of solutions of MV^2+^2I^−^ and CB[7], indicating the adsorption of I^−^, i.e., the counter anions of MV^2+^2I^−^, on Au NPs surface (Fig. 3a). It was further confirmed by the scanning transmission electron microscopy (STEM) elemental mapping of Au NPs aggregates. In the STEM images shown in Fig. 3b, a uniform distribution of I^−^ was observed over Au NPs surface. It has been reported that halide ions can specifically adsorbed on gold surface^37,38^, thereby facilitating the co-adsorption of positively charged molecules through electrostatic interactions^39,40^. Therefore, we conjectured that there exsits the cooperative adsorption among counter anions, MV^2+^, and CB[7]. Specifically, the halide counter anions are directly adsorbed on Au NPs surface and attract MV^2+^; then, CB[7] interacts with the adsorbed MV^2+^ to form a sandwich structure.

To verify this conjecture, I^−^ was first replaced with Br^−^, Cl^−^, and ClO_4_^−^ with the binding affinity to Au surface varying in the following order: I^−^>Br^−^>Cl^−^>>ClO_4_^−^^37,38^. As shown in Fig. 3c, the SERS spectrum for MV^2+^2Br^−^ is similar to the case of MV^2+^2I^−^. In comparison, no SERS signals were observed for MV^2+^2Cl^−^ and MV^2+^2ClO_4_^−^ even at a fourfold increased concentration of guest or host molecules (Fig. S8a). In addition, by replacing MV^2+^ cations with Na^+^ cations that also bind to CB[7]^41^, Cl^−^ anions still did not work at the same concentration when compared to I^−^ and Br^−^ anions (Fig. S8b). Thus, the adsorption of halide counter anions on the surface played a key role in the aggregation of Au NPs.

Second, the replacement of MV^2+^2I^−^ with NaI induced no SERS signal even at a fourfold increased concentration of Na^+^ and I^−^ up to 12.5 μM (Fig. 3d), indicating the key role of MV^2+^ cations in the aggregation of Au NPs.

### Interfacial CB[7]-MV^2+^ complexation

**Fig. 4:**
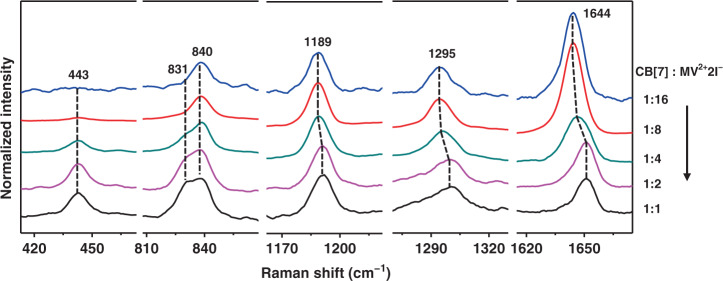
Normalized SERS spectra (1189 cm^−1^ peak of MV^2+^ as standard) in ascending order of molar ratio of CB[7]:MV^2+^2I^−^ from top to bottom. The concentration of MV^2+^2I^−^ was kept constant at 2.5 μM, and that of CB[7] varied at 0.16 μM, 0.31 μM, 0.62 μM, 1.25 μM, and 2.5 μM from top to bottom

To further investigate the host–guest complexation at the interface, the CB[7] concentration-dependent SERS experiments were conducted. As shown in Fig. 4, the increase in molar ratio of CB[7]:MV^2+^2I^−^ from 1:8 to 1:2 led to enhanced Raman signals of CB[7], including the peaks at 443 cm^−1^ and ca. 831 cm^−1^. Besides, the relative intensity of 1644 cm^−1^ peak from MV^2+^ decreased significantly. Moreover, the MV^2+^ peaks at 1189 cm^−1^, 1295 cm^−1^, and 1644 cm^−1^ gradually shifted to 1192 cm^−1^, 1301 cm^−1^, and 1651 cm^−1^, respectively. Interestingly, such peak shifts were not observed for the same host–guest systems in homogenous solutions (Fig. S4), suggesting the occurrence of a distinct host–guest complexation mechanism on the surface.

**Fig. 5:**
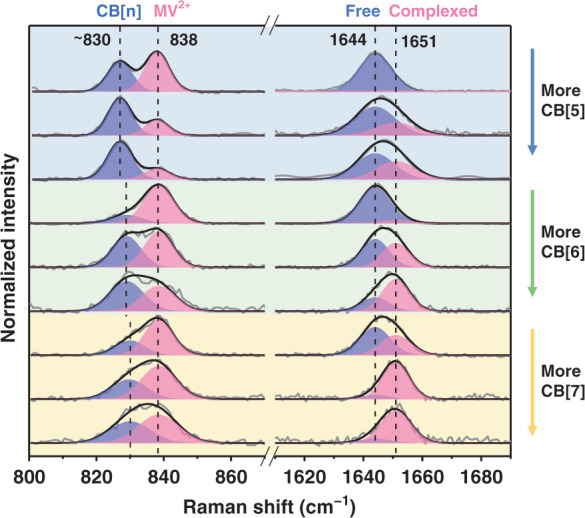
CB[5]/CB[6]/CB[7] concentration-dependent SERS spectra obtained with 2.5 μM MV^2+^2I^−^. The concentrations of CB[5]/CB[6]/CB[7] are 0.62 μM, 1.25 μM, and 2.5 μM from top to bottom

To better understand the complexation mechanisms of CB[7] and MV^2+^2I^−^ at the interface, control experiments were performed by replacing CB[7] with other cucurbiturils of smaller cavity sizes, including CB[5] and CB[6]. Unlike CB[7], smaller host molecules like CB[5] and CB[6] cannot encapsulate bipyridinium owing to their smaller portal diameters and cavity sizes^4^. Hence, they only form exclusion complexes with MV^2+^ at the portals but not inside the cavities (Fig. S9). Interestingly, both CB[5] and CB[6] also cooperatively induced Au NPs aggregation with MV^2+^2I^−^ (Fig. S10). Moreover, similar blue shifts in the MV^2+^ peak at 1644 cm^−1^ were also observed for CB[5] and CB[6] systems (Fig. 5). The shifts resulted from the gradual conversion of 1644 to 1651 cm^−1^ peak, as shown by the Gaussian function fitting. The two peaks were assigned to the adsorbed MV^2+^2I^−^ complexed without (Fig. S11) or with (Fig. S12) CB[n], respectively. Therefore, the complexation model of CB[7] and MV^2+^2I^−^ at Au NPs surface may be analogous to that of CB[5] and CB[6] systems. In other words, CB[7] and MV^2+^2I^−^ formed an exclusion complex on Au NPs surface instead of a conventional 1:1 inclusion complex. Furthermore, the relative Raman intensity of 1651 cm^−1^ peak over 1644 cm^−1^ peak under the same conditions increased in the following order: CB[5]<CB[6]<CB[7] system. The quantitative analysis of complexation (Fig. S14) shows a similar 1:2 complexation model between CB[5]/CB[6]/CB[7] and MV^2+^2I^−^ at the interface, regardless of the changed inner size of CB[n], and the obtained interfacial binding constants of host molecules on the surface increase in the following order: CB[5]<CB[6]<CB[7].

**Scheme 1:**
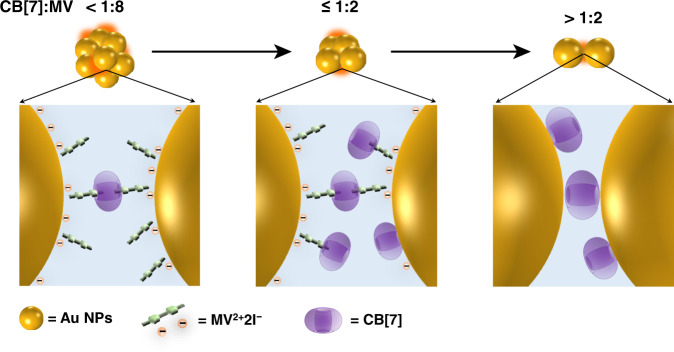
A proposed mechanism for the interfacial interaction among I^−^, MV^2+^, and CB[7]

Based on these data, an exclusion complexation model between CB[7] and MV^2+^2I^−^ at a solid–liquid interface was constructed as shown in Scheme 1. With the aid of electrostatic attraction of adsorbed I^−^, positively charged MV^2+^ was adsorbed onto the Au NPs surface in a near-vertical fashion. Meanwhile, the dangling positive N atom in MV^2+^ interacts with the portal of CB[7] and the methyl group is encapsulated in the cavity of CB[7]. This host–guest interaction could be driven by ion-dipole interactions between the positive N atom and negative portal of CB[7], as well as the hydrophobic force between methyl group and cavity of CB[7]^29,42,43^. The interfacial interactions among Au NPs, CB[7], and MV^2+^2I^−^ were also regulated by the stoichiometry of CB[7] to MV^2+^2I^−^, relevant to Au NPs aggregation (Figs. S15 and S16). Precisely, a low stoichiometry of CB[7] (CB[7]:MV^2+^2I^−^≤1:8) already caused the intense aggregation of Au NPs (Scheme 1, left). The Au NPs might be bridged by CB[7]-MV^2+^ complexes in the form that one CB[7] molecule binds two adsorbed MV^2+^ molecules from adjacent Au NPs. As the content of CB[7] increased (1:8 < CB[7]:MV^2+^2I^−^≤1:2), more CB[7] would bind to the adsorbed MV^2+^. This results in electrostatic repulsion among Au NPs and partially cleavage of the linkers, thereby reducing the aggregation of Au NPs (Scheme 1, middle). Meanwhile, CB[7] is directly adsorbed on Au NPs surface and compete against the adsorption of MV^2+^2I^−^. Excess CB[7] (CB[7]:MV^2+^2I^−^ > 1:2) would completely inhibit the adsorption of MV^2+^2I^−^ on Au NPs surface, thereby eliminating the cooperative effect between MV^2+^2I^−^ and CB[7] and further reducing the aggregation of Au NPs (Scheme 1, right).

## Discussion

The host–guest complexation at the solid–liquid interface is usually assumed to be the same as that in homogeneous solutions. However, synergistic effects among multiple noncovalent interactions based on electrostatic, hydrophilic, and hydrophobic interactions combined with the surface effects like steric hindrance and competitive adsorption^5,24^ greatly affect the host–guest complexation on the surface. Here, the SERS results suggest an unconventional host–guest complexation model on the surface, distinct from the well-known 1:1 inclusion complexation model in aqueous solutions.

Cooperative adsorption of halide counter anions, MV^2+^, and CB[7]occurred on the Au NPs surface. The adsorbed I^−^ anions on Au NPs surface promoted the gathering of MV^2+^ cations, as well as the host molecules. Under the condition, one positively charged N atom of MV^2+^ interacted with I^−^ anions present on the surface, which may impede the encapsulation of bipyridinium unit of MV^2+^ by CB[7] owing to the electrostatic repulsion between I^−^ anions and carbonyl oxygens at the CB[7] portal. DFT calculation (Fig. S17) shows a different profile in the case with I^−^ compared with that without I^−^; the two local minimums are both exclusive complexation models, suggesting a significant effect of counter anions on interfacial host–guest complexation.

The intrinsic weakness in physical adsorption^44^ led to inevitable competitive adsorption among the host, guest, and counterions on the surface. Competitive adsorption on the surface resulted in diverse surface environments, thereby affecting the host–guest complexation behavior on the surface. Here, the competitive adsorption between I^−^ and CB[7] on Au NPs surface was clearly characterized by SERS, and it was verified to be closely relevant to the aggregation Au NPs.

In summary, the interfacial interactions of CB[n]-based host–guest system on Au NPs surfaces were systematically studied by SERS. The ultrahigh sensitivity and rich molecular vibrational information provided by SERS allowed the determination of unreported cooperative adsorptions among counter anions (I^−^ and Br^−^), guest cations (MV^2+^ and Na^+^) and hosts (CB[5], CB[6], and CB[7]) on the surface. Moreover, an exclusion complex model between CB[7] and MV^2+^ distinct from that in aqueous solutions was proposed based on the SERS results. These findings provide new insights into the fundamental understanding of host–guest interactions at the solid–liquid interface, promising for applications in host–guest chemistry for engineered nanomaterials.

## Materials and methods

### Reagents

Methyl viologen diiodide (MV^2+^2I^−^) and methyl viologen dichloride (MV^2+^2Cl^−^) were purchased from J&K Chemical. CB[7], CB[6], and sodium 3-(trimethylsilyl)-1-propanesulfonate [^1^H NMR standard used for D_2_O solvent] were obtained from Sigma-Aldrich. Methyl viologen dibromide (MV^2+^2Br^−^) was synthesized following literature^45^. High-purity water (Milli-Q, 18.2 MΩ cm) was used throughout the studies.

### Preparation of Au NPs

In all, 50 nm Au NPs were prepared using the citrate reduction method reported by Lee and Meisel^46^. To concentrate the Au NPs by 10 times, 14 mL colloid suspension was centrifuged (3500 rpm, 15 min) once and resuspended in 1.4 mL H_2_O after the removal of all the supernatant.

### Preparation of stock solutions

All stock solutions (2 mM) were prepared in 4 mL H_2_O followed by dilution to 50 μM and then to 10 μM.

### UV-Vis spectroscopy

The ultraviolet-visible (UV−vis) spectra were recorded using a Shimada UV-2550 spectrophotometer. In all, 100 µL Au NPs and 300 µL of reagents were mixed and immediately placed in a cuvette with a one-millimeter optical path. The time-dependent spectra were acquired at 1 min intervals for 5 min.

### ζ-potential measurements

The ζ-potential was collected using a Malvern Zetasizer Nano ZS Instrument. First, 200 µL Au NPs and 600 µL reagents were mixed and then immediately placed into a cuvette. The temperature was set as 25°C, and every sample was tested three times.

### SERS experiments

The SERS data were collected using a Renishaw Invia instrument (5 mW, 633 nm, ×50 objective, NA = 0.55, 1800 gr/mm grating, and 20 s per scan). First, calculated volumes of stock solutions were mixed in a 96-well plate, and then 50 µL of concentrated Au NPs was added and mixed through pipetting a dozen times. The total volume was adjusted to 200 µL, and SERS measurements were immediately carried out after mixing by focusing the laser beam directly on the sample suspension.

### Transmission electron microscopy

TEM images were viewed using a JEM-1400 (JEOL, Japan) and scanning TEM images were collected using a Talos F200 instrument (FEI, USA).

### Nuclear magnetic resonance

NMR spectra were collected using a Bruker AVANCE III-500 MHz NMR unless otherwise stated.

## Supplementary information

Supplementary information
