# Supplementary material for: Revealing unconventional host–guest complexation at nanostructured interface by surface-enhanced Raman spectroscopy
Source: Light Sci Appl. 2021 Apr 19;10:85. doi: 10.1038/s41377-021-00526-5 (PMC8055983; doi:10.1038/s41377-021-00526-5)
Supplement: Supplementary file 1 — Supplementary information [file 41377_2021_526_MOESM1_ESM.docx]

**Supplementary Information for**

**Revealing Unconventional Host-Guest Complexation at Nanostructured Interface by Surface-Enhanced Raman Spectroscopy**

Gan-Yu Chen^a^, Yi-Bin Sun^a^, Pei-Chen Shi^a^, Tao Liu^ab^, Zhi-Hao Li^a^, Si-Heng Luo^ab^, Xin-Chang Wang^c^, Xiao-Yu Cao^ad^, Bin Ren^a^, Guo-Kun Liu^*,b^, Liu-Lin Yang^*,a^, Zhong-Qun Tian^*,a^

^a^ State Key Laboratory of Physical Chemistry of Solid Surfaces, Collaborative Innovation Center of Chemistry for Energy Materials (*i*ChEM), College of Chemistry and Chemical Engineering, Xiamen University, Xiamen, 361005, China

^b^ State Key Laboratory of Marine Environmental Science, Fujian Provincial Key Laboratory for Coastal Ecology and Environmental Studies, Center for Marine Environmental Chemistry & Toxicology, College of the Environment and Ecology, Xiamen University, Xiamen 361102, China

^c^ School of Electronic Science and Engineering (National Model Microelectronics College), Xiamen University, Xiamen 361005, China

^d^ Key Laboratory of Chemical Biology of Fujian Province, Xiamen University, Xiamen 361005, China

AUTHOR INFORMATION

Corresponding Author

* Emails: guokunliu@xmu.edu.cn, llyang@xmu.edu.cn, zqtian@xmu.edu.cn

**Table S1**. ζ-potential measurement results

**
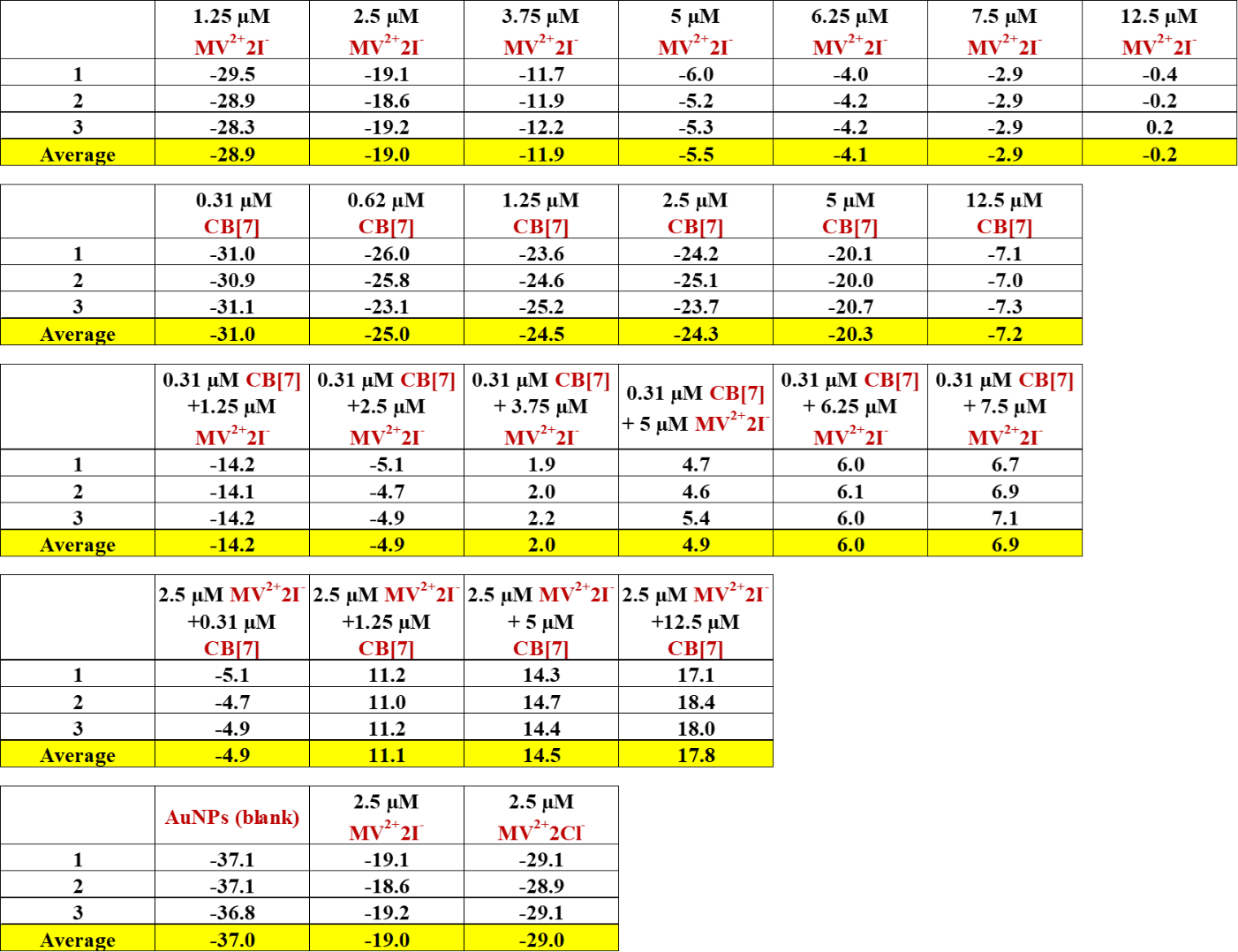
**

**
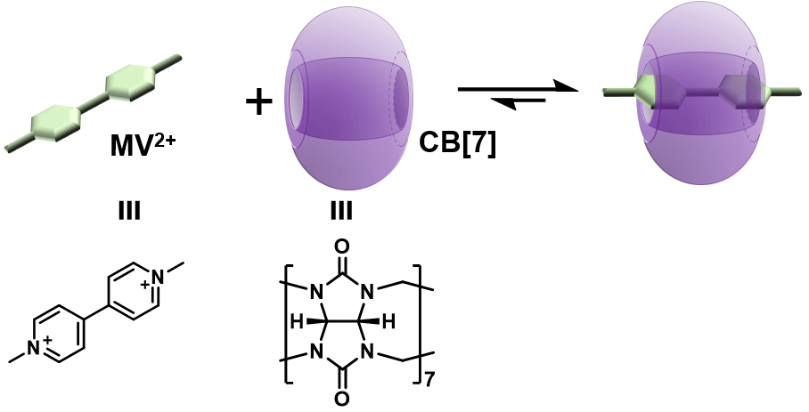
**

**Figure S1.** The 1:1 inclusion complex between CB[7] and MV^2+^2I**^−^** in aqueous solution.


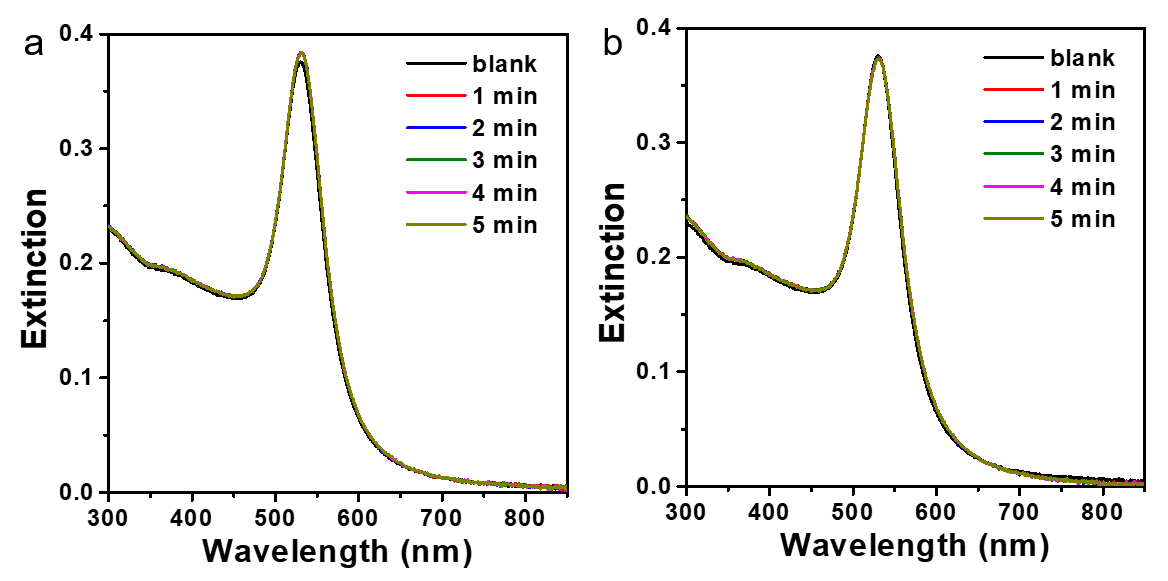


**Figure S2.** Time-dependent extinction spectra recorded immediately after the addition of the solution of a) MV^2+^2I**^−^** (2.5 μM) and b) CB[7] (0.31 μM) within 5 min. Note: The spectra were acquired at 1 min intervals.


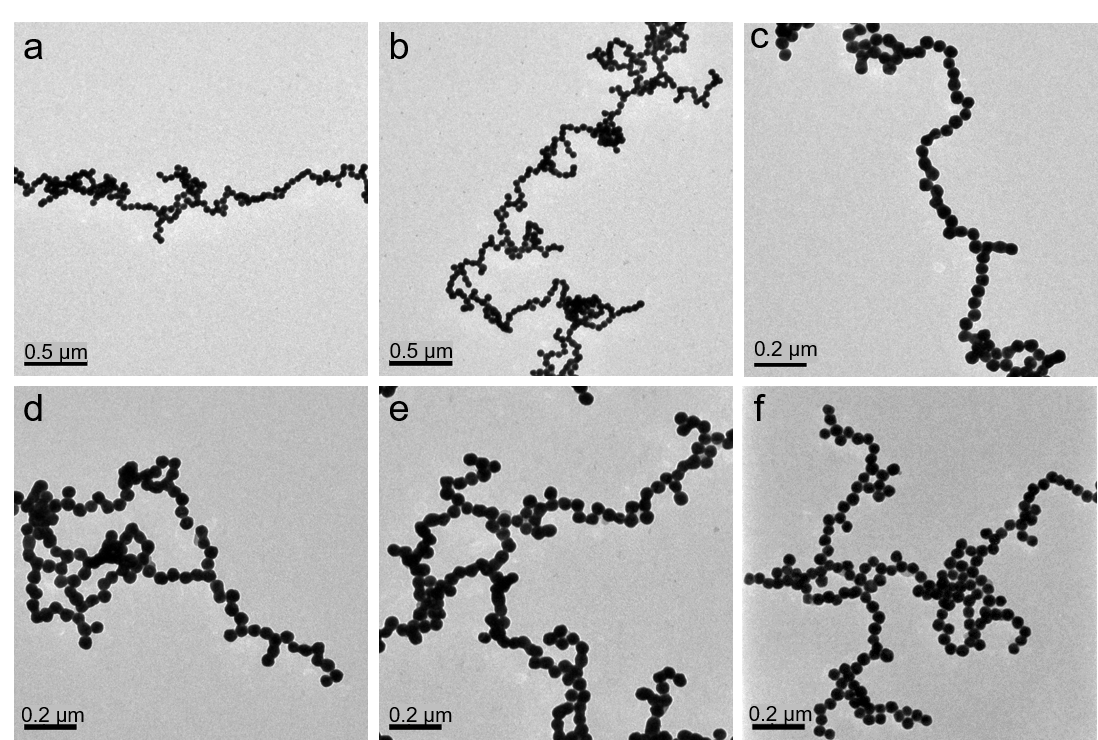


**Figure S3**. An interesting and orderly self-assembly phenomenon was observed with the formation of linear Au NPs aggregates when the ζ-potential approach to zero, namely when the concentration of CB[7] were 0.31 μM with sufficient MV^2+^2I**^−^** (2.5 μM or more) (Figure 2a). (a-e) TEM images of the aggregates of Au NPs induced by the solution of MV^2+^2I^-^ (25 μM) + CB[7] (0.31 μM). f) TEM image of the aggregates of Au NPs induced by the solution of MV^2+^2I**^−^** (2.5 μM ) + CB[7] (0.31 μM).


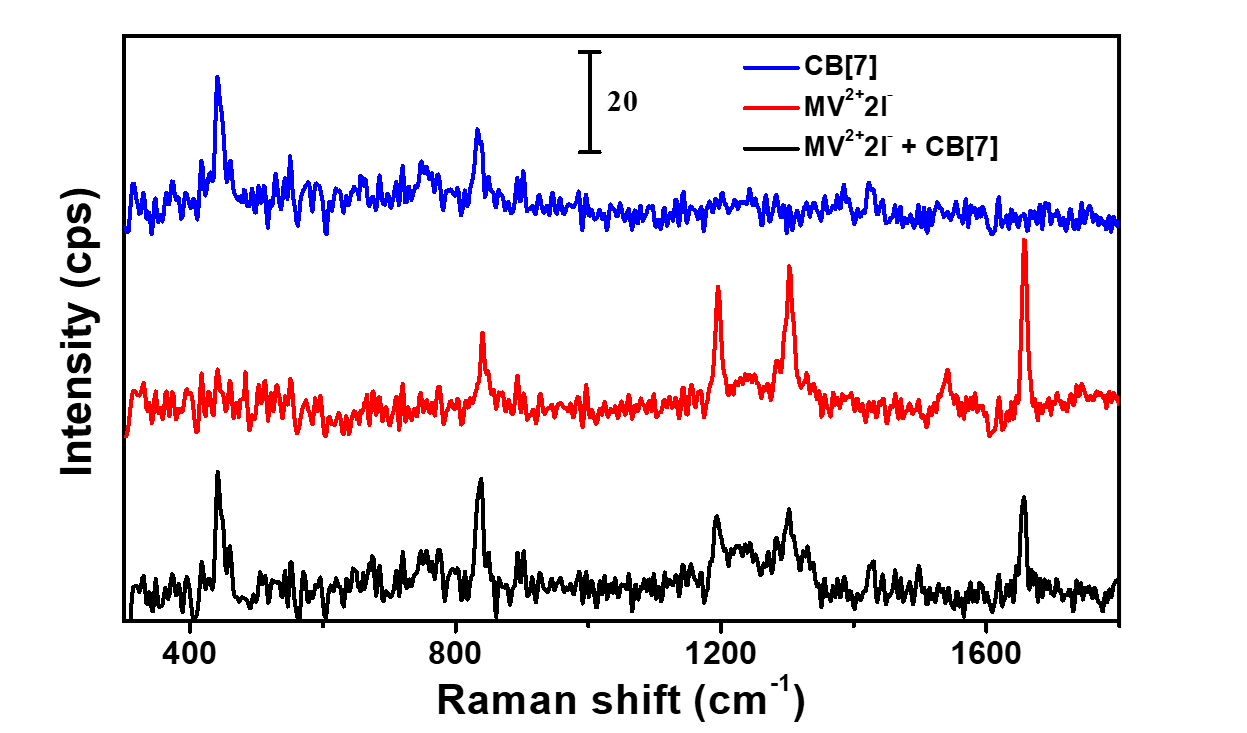


**Figure S4.** Raman spectra of CB[7] (2.5 mM), MV^2+^2I**^−^** (2.5 mM) and MV^2+^2I**^−^** (2.5 mM) + CB[7] (2.5 mM) in aqueous solution.


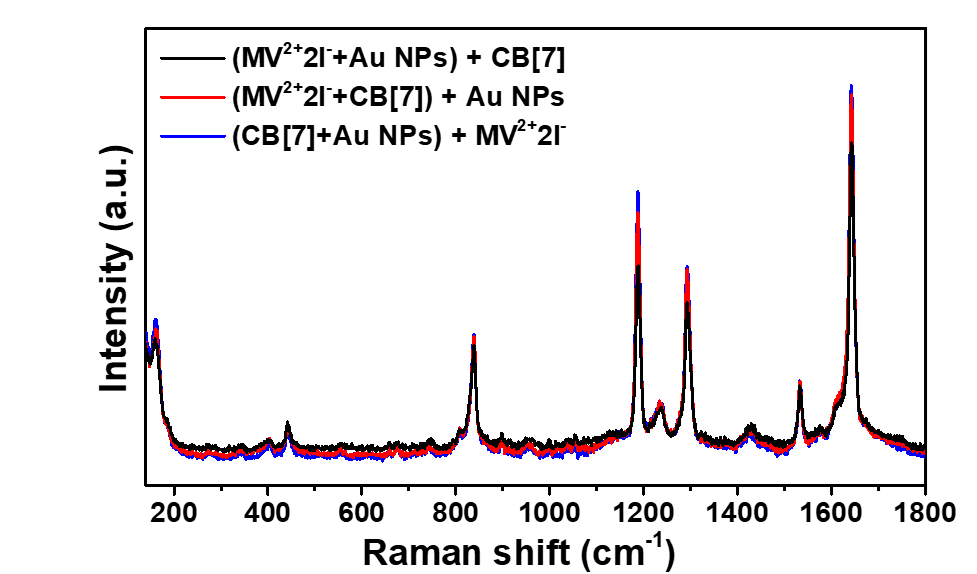


**Figure S5.** Mixing order dependent SERS spectra for the case of CB[7], MV^2+^2I^−^ and the colloidal Au NPs. The concentrations of MV^2+^2I^−^ and CB[7] were 2.5 μM and 0.31 μM, respectively. The aggregation of Au NPs and SERS performance are independent of the mixing order of CB[7], MV^2+^2I^−^ and the colloidal Au NPs, evidenced by the almost identical (relative) SERS intensities and peak locations.


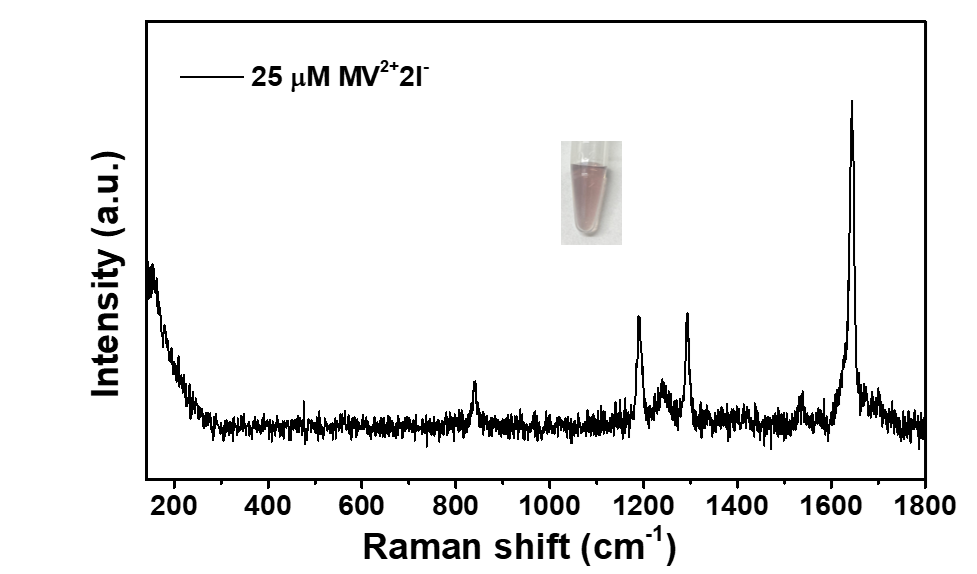


**Figure S6.** The SERS spectra of Au NPs colloids upon addition of 25 μM MV^2+^2I^−^. Inset showing the corresponding photograph of Au NPs colloid. MV^2+^2I^−^ alone can also induce the aggregation of Au NPs, but only with a higher concentration than the ones applied in the host-guest induced aggregation. The results verified the cooperative effect between host and guest in aggregating Au NPs.


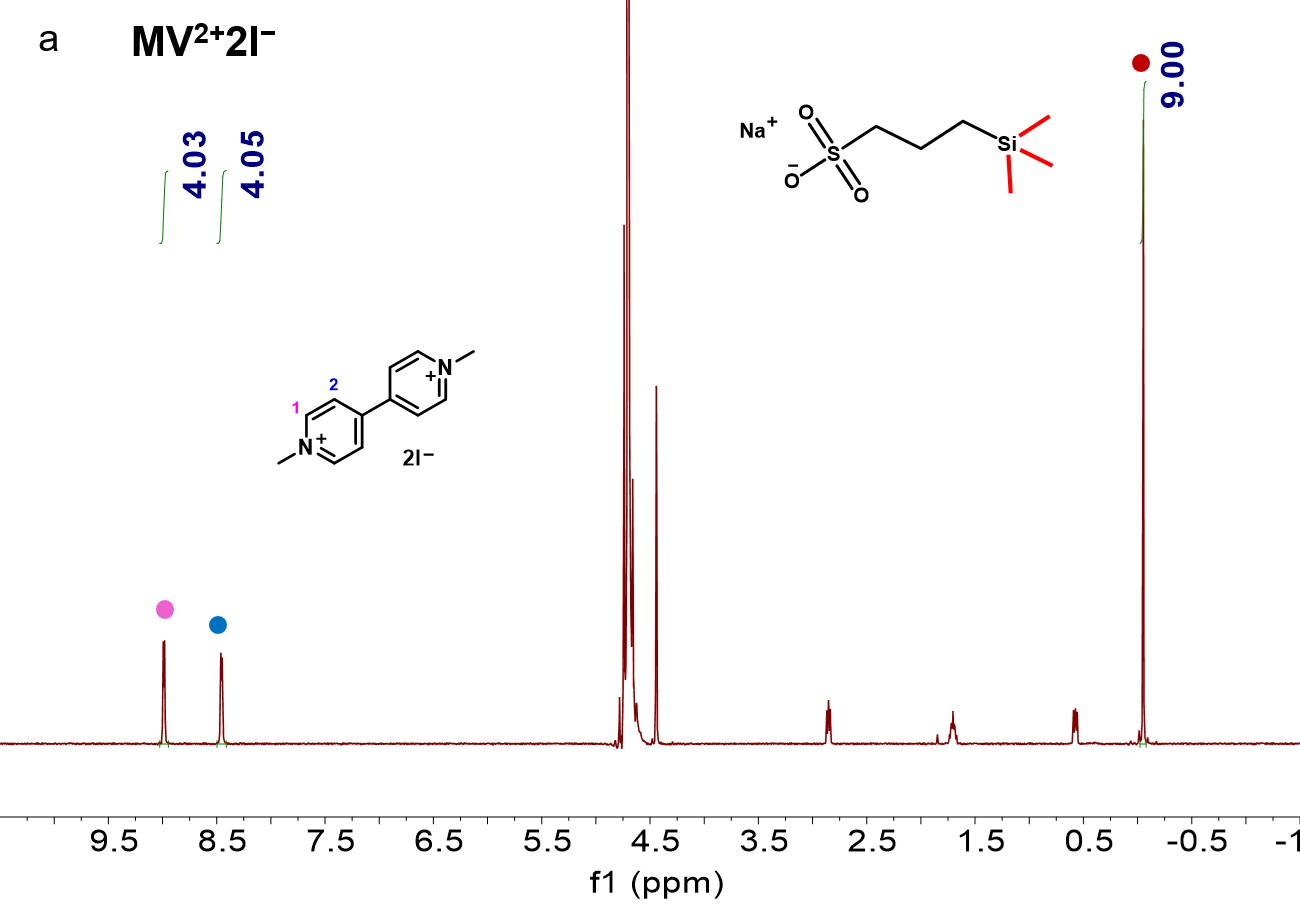


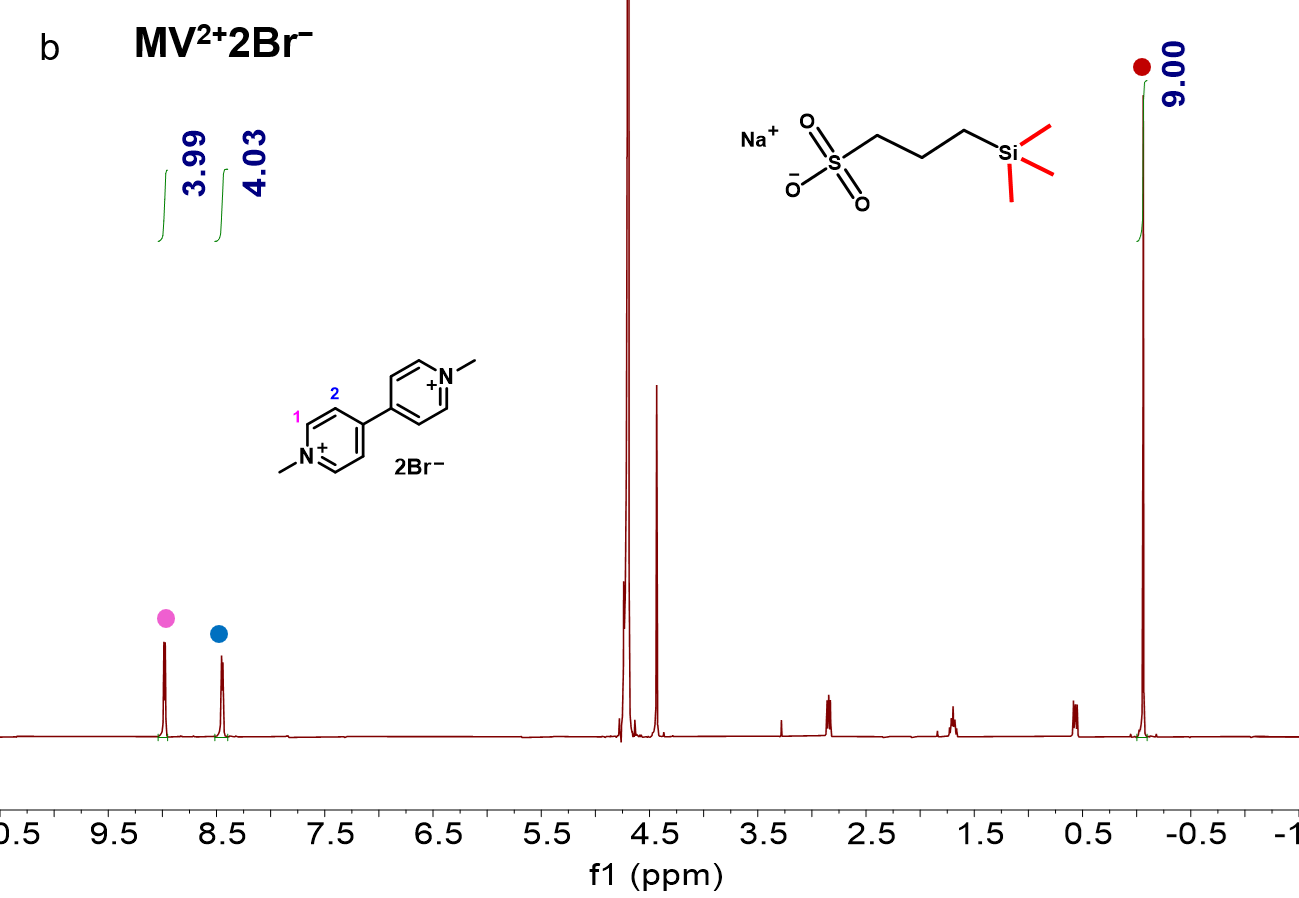


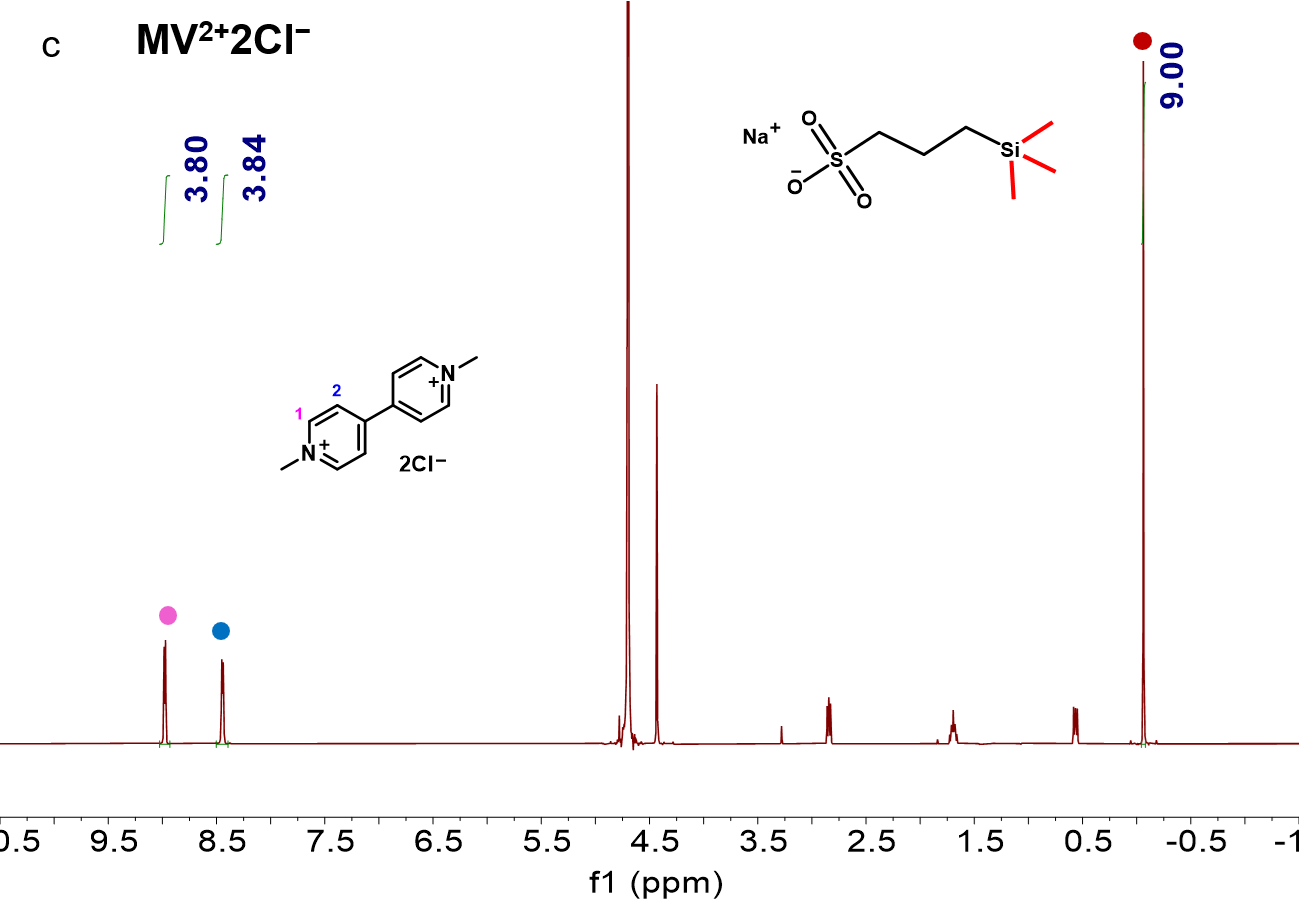


**Figure S7.** NMR concentration calibration results of a) MV^2+^2I**^−^**, b) MV^2+^2Br**^−^**, and c) MV^2+^2Cl**^−^** with sodium 3-(Trimethylsilyl)-1-propanesulfonate as the ^1^H NMR standard in D_2_O. The purity of three halide salts were all > 95%.


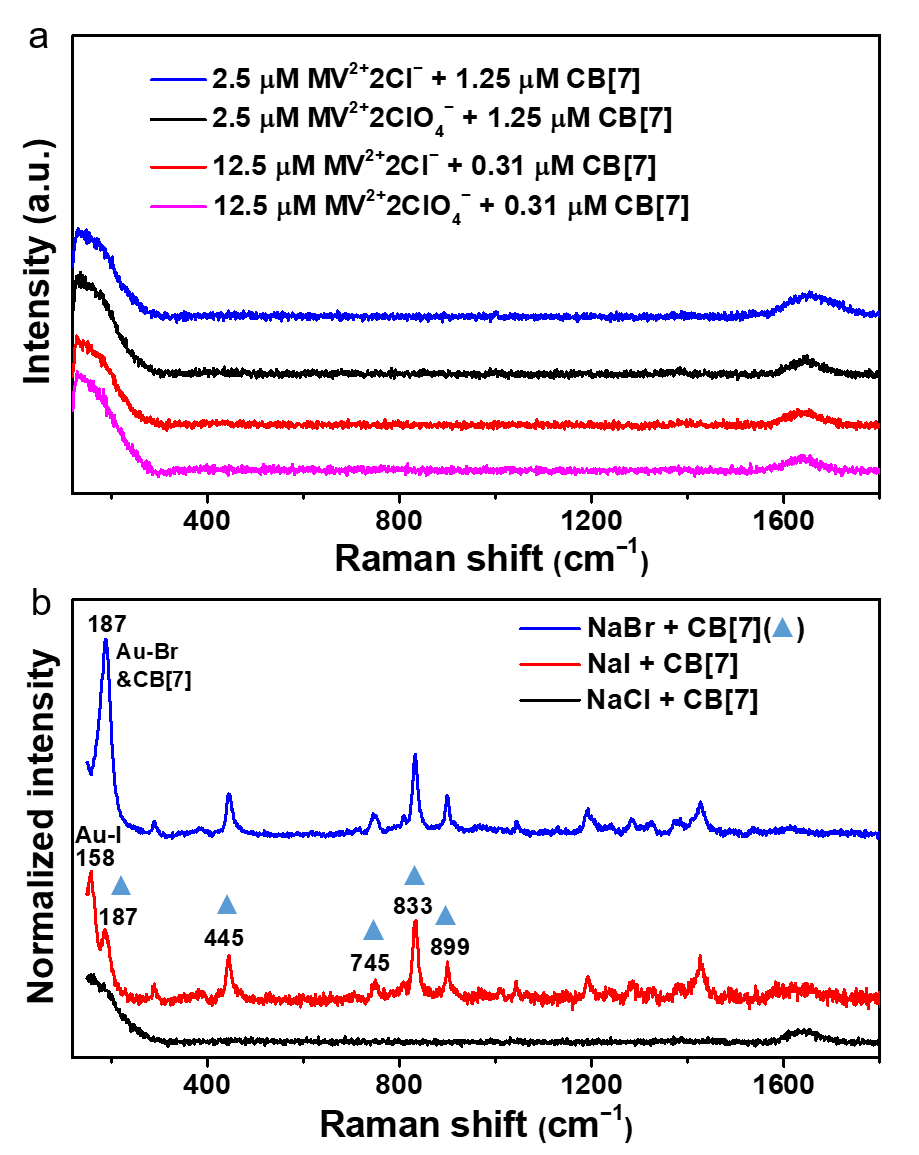


**Figure S8.** a) SERS spectra of colloidal suspensions when increased the concentration of guest or host molecules by five times; b) SERS results of the variable-controlling experiment in which only the type of halide ions brought by sodium salt was different. The concentration of CB[7] and all the sodium salt were 1.25 μM and 2.5 μM, respectively.


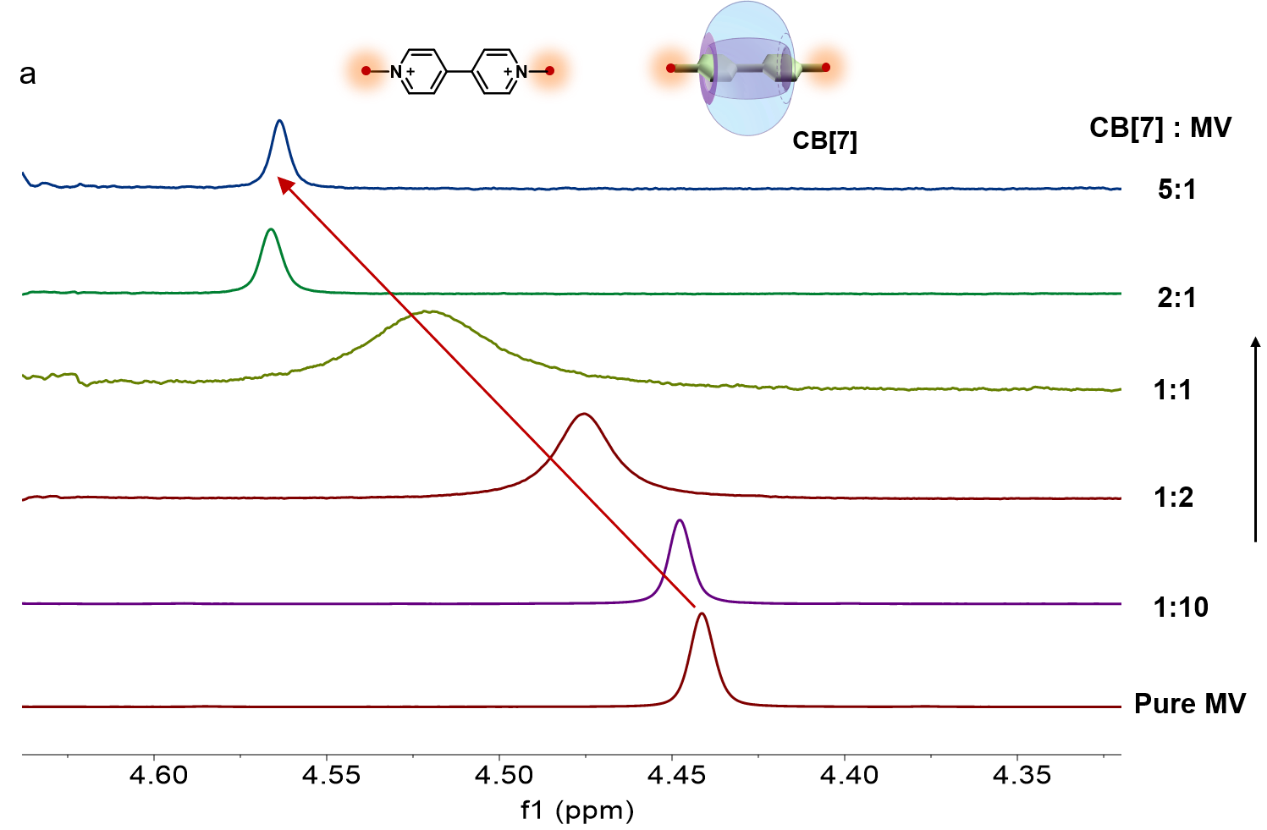


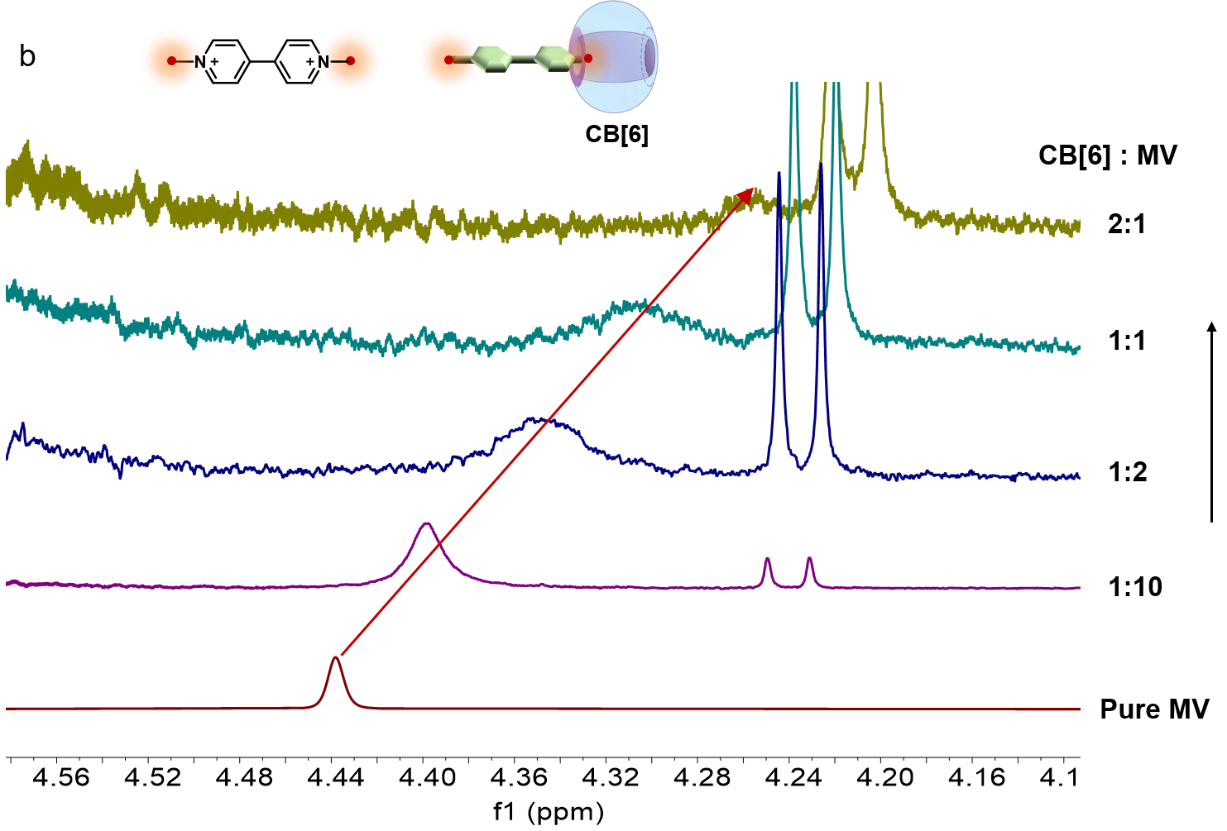


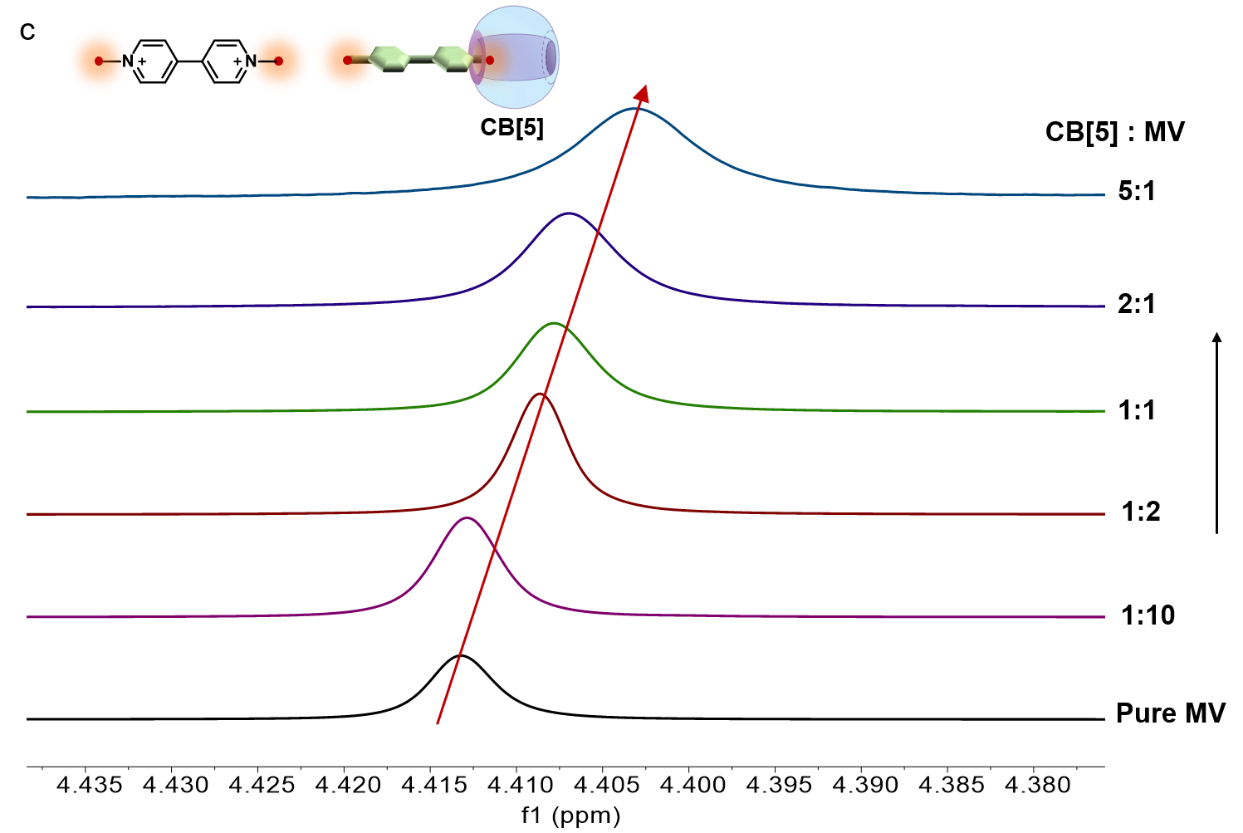


**Figure S9.** ^1^H NMR spectra of MV^2+^ in the presence of different concentration of a) CB[7], b) CB[6] (850 MHz) and c) CB[5] in D_2_O. The methyl protons of MV^2+^ experienced an upfield shift with the increase of CB[5]/CB[6], in contrast to the downfield shift for CB[7] case, suggesting the exclusive complexation between CB[5]/CB[6] and MV^2+^ in aqueous solution, where the methyl groups were included within their cavities.


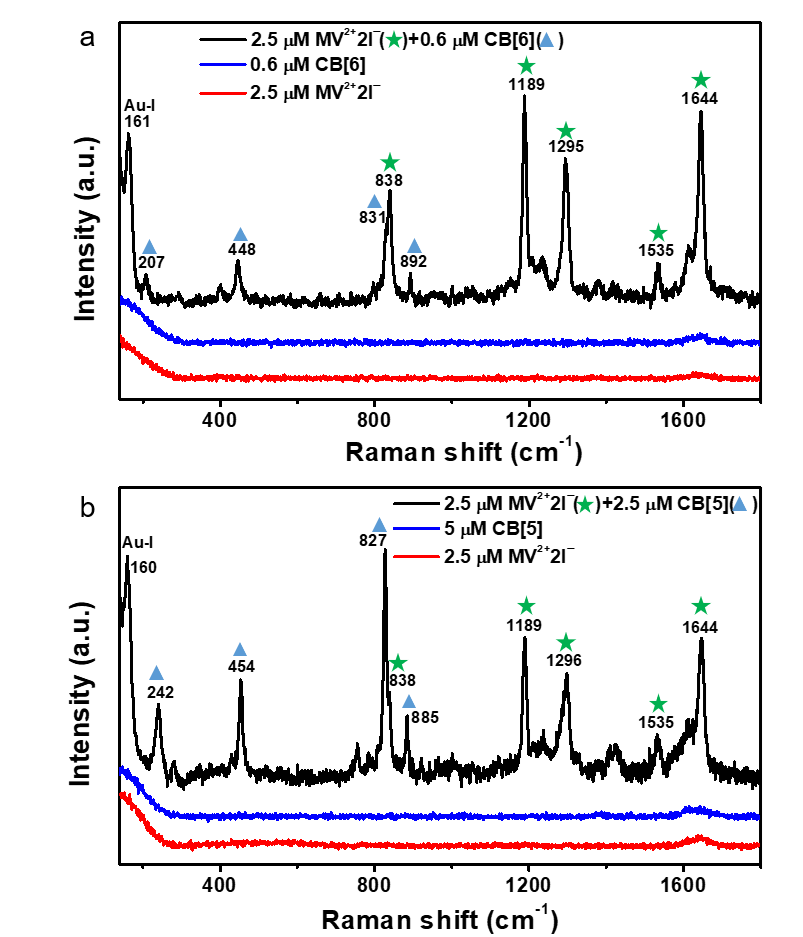


**Figure S10.** SERS spectra of Au NPs colloids upon addition of MV^2+^2I**^−^** + CB[6]/CB[5], MV^2+^2I**^−^** and CB[6]/CB[5], respectively.


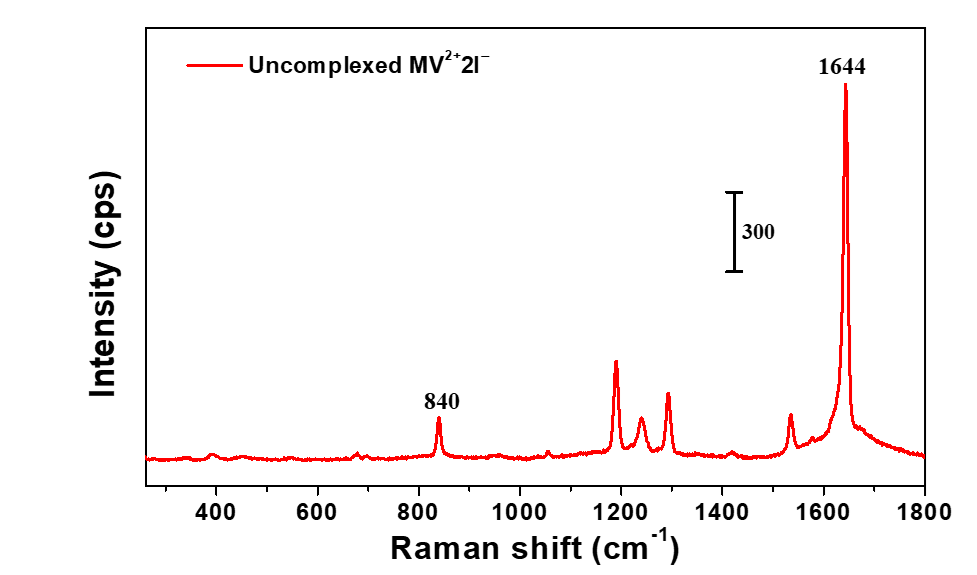


**Figure S11.** SERS spectra of Au NPs colloids upon addition of NaI (1.25 mM) + MV^2+^2I**^−^** (2.5 μM). 1644 cm^−1^ peak is also observed in the SERS spectra in the controlled experiment that NaI and MV^2+^2I^−^ were employed as co-aggregator without CB[n], indicating this peak comes from the adsorbed MV^2+^ interact with I^−^ without complexing with CB[n]. The results also verified the adsorbed I^−^ can facilitate the co-adsorption of positive charged molecules through electrostatic interactions.

**
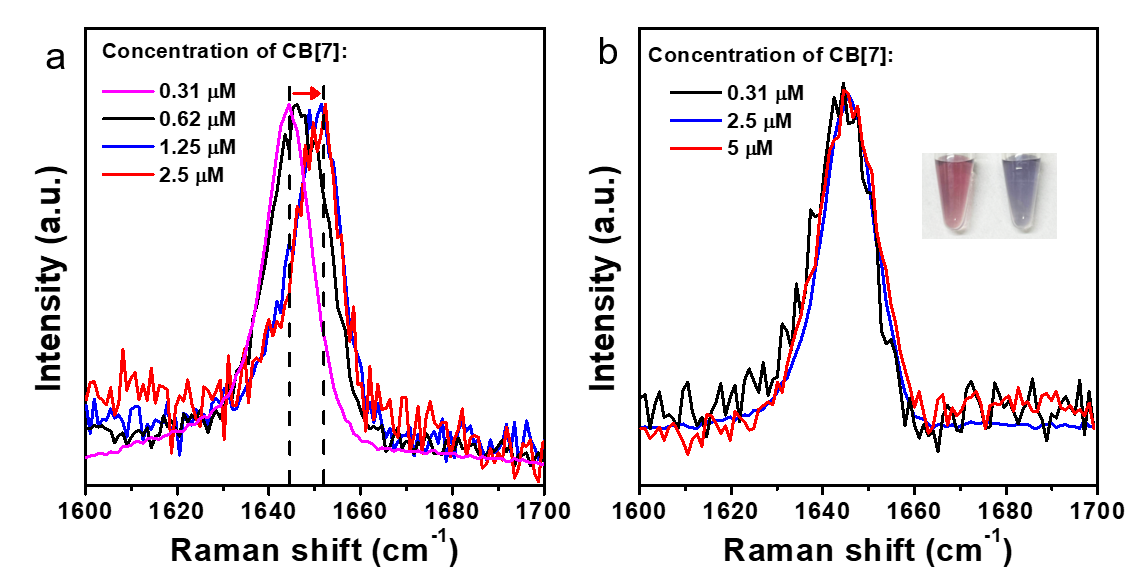
**

**Figure S12.** SERS spectra show the upshifts of MV^2+^ band at 1644 cm**^−^**^1^ and no shift in the (a) absence and (b) presence of 1.25 mM NaI, respectively. Inset shows the corresponding photographs of Au NPs colloids (from left to right) upon the addition of 0.31 μM and 2.5 μM CB[7] respectively. The concentration of MV^2+^2I**^−^** kept constant as 2.5 μM. The 1644 cm^−1^ peak did not experience any shift with the increase of CB[7] in the presence of higher concentration of NaI, suggesting excess of Na^+^ hinders the host-guest complexation between CB[7] and MV^2+^ since the competitive complexation of Na^+^ with the portals of CB[7]^1^. Besides, the corresponding photographs of Au NPs colloids in insets indicate that the different aggregation state could not induce the SERS peak shifts. All the results confirm that the 1651 cm^−1^ peak indeed comes from the complexed MV^2+^ with CB[n].


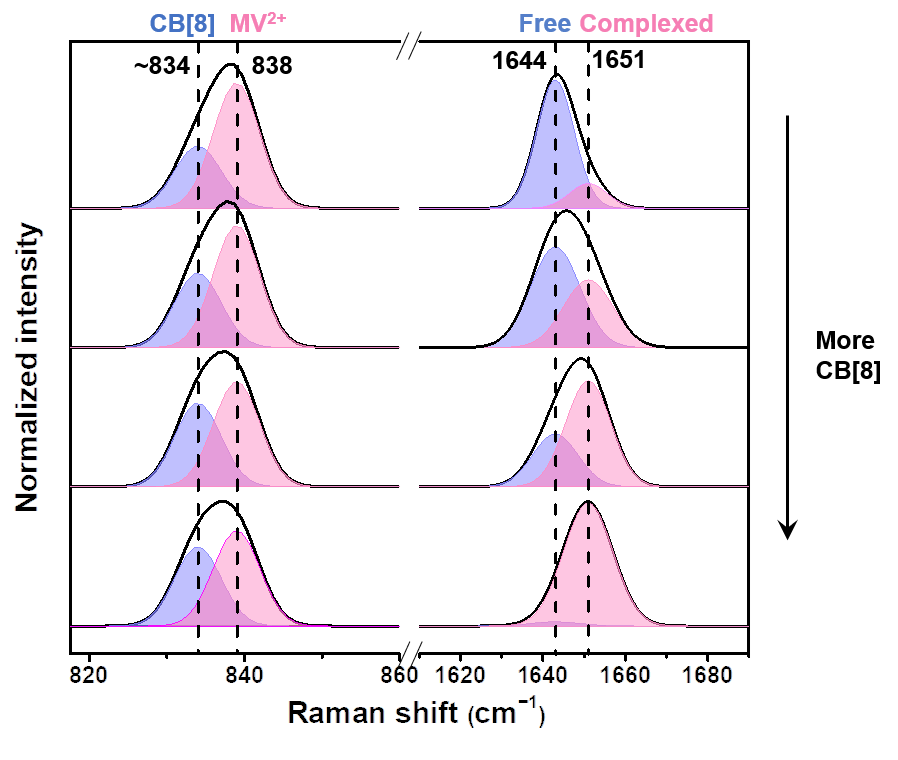


**Figure S13.** CB[8] concentration-dependent normalized SERS spectra with 2.5 μM MV^2+^2I^−^. The concentration of CB[8] was 0.31 μM, 0.62 μM, 1.25 μM and 2.5 μM from top to bottom. The results show that the complexation of MV^2+^2I^−^ with CB[8] of which the cavity is larger than CB[7], also experiences the similar blue shift as CB[5]/CB[6]/CB[7], indicating that CB[8] and MV^2+^2I^−^ also form an exclusion complex on Au NPs surface.


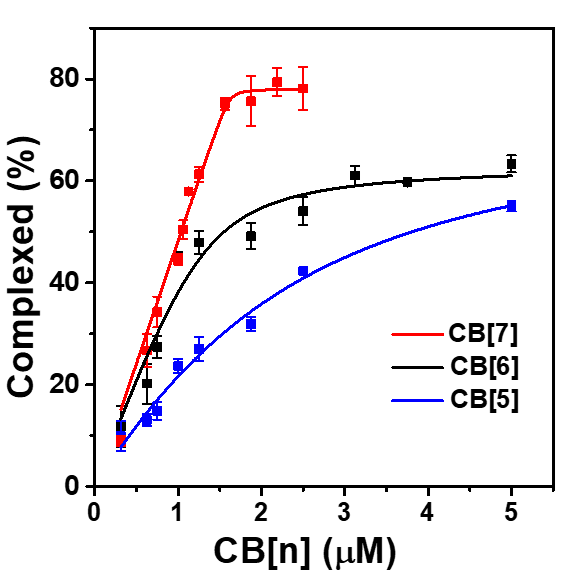


**Figure S14.** Plots of the peak area proportion of complexed MV^2+^ at 1651 cm^-1^ in the sum of 1644 and 1651 cm^-1^ peak area as a function of increasing CB[n]s (n=5,6 and 7) concentration, and the fitted profile fitting the peak area proportion with a one-sites binding model:

$$a=d*\frac{mKx+c_{0}K+1-\sqrt{\left( mKx+c_{0}K+1 \right)^{2}-{4c_{0}mK}^{2}x}}{2c_{0}K}$$

where $K$ represents the binding constant, $a$ represents the conversion ratio, $d$ represents the maximum conversion ratio, $m$ represents the stoichiometric ratio, $c_{0}$ represents the initial concentration.

Accordingly, the values of *m*, i.e. the stoichiometric ratios between CB[5/6/7] and MV^2+^2I^−^, all approximately equal to 2, suggesting a similar 1:2 complexation model between CB[5/6/7] and MV^2+^2I^−^ at interface. Furthermore, the binding constants ($K$) of CB[7], CB[6] and CB[5] are 3.9*10^8, 4.0*10^6, and 4.1*10^5 M^-1^, respectively. The complexation affinity of CB[7] and MV at interface is about an order of magnitude larger than 1.3*10^7 in aqueous solution,^2^ which might be caused by the preorganization of ligands on surface as reported by previous studies.^3^


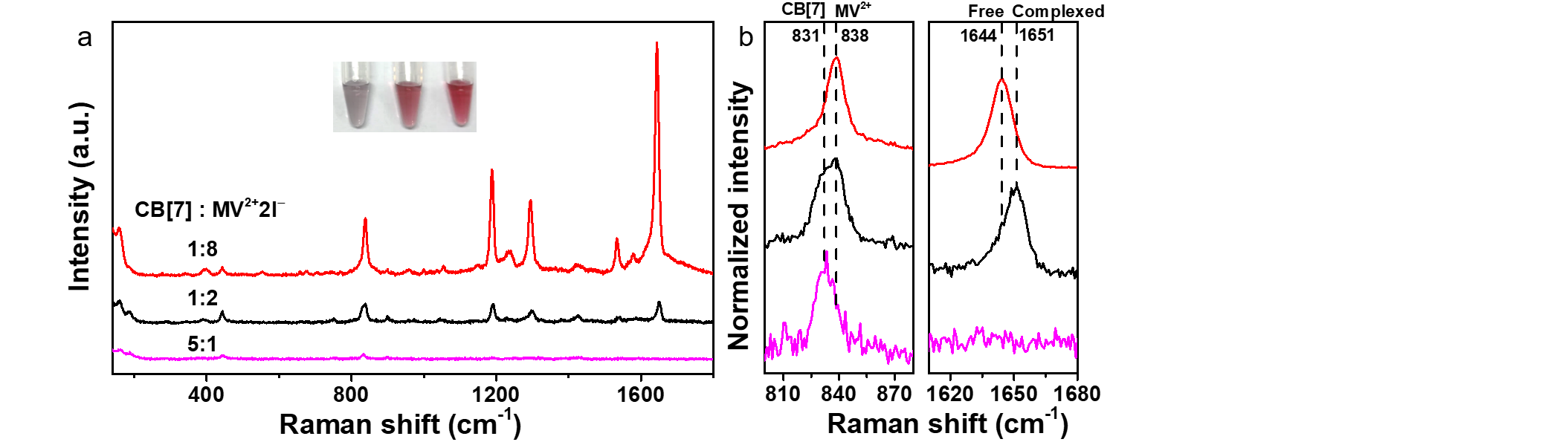


**Figure S15.** a) The original and b) the normalized SERS spectra of Au NPs colloid upon addition of different concentration of CB[7] with 2.5 μM MV^2+^2I^−^. Inset showing the corresponding photographs of Au NPs colloid (from left to right) upon addition of the CB[7]-MV^2+^2I^−^ mixture with the proportion of 1:8, 1:2 and 5:1, respectively. The interfacial interactions among Au NPs, CB[7], and MV^2+^2I^−^ were altered by the ratio of CB[7] to MV^2+^2I^−^, relevant to Au NPs aggregation. When the ratio of CB[7] and MV is 1:8, the strongest SERS signal was observed (Figure S14a, red line), and the dominated 1644 cm^-1^ peak from free MV^2+^ (Figure S14b, red line) indicates that the majority of MV^2+^ in the SERS hot spots region is uncomplexed. As the ratio increases to 1:2, the SERS intensity significantly decreases (Figure S14a, black line), originating from the less aggregation of Au NPs (the inset of Figure S14a). Meanwhile, the dominated 1651 cm^-1^ peak from complexed MV^2+^ (Figure S14b, black line) indicates that the majority of MV^2+^ in the hot spots region is complexed. When the ratio further increases to 5:1, only the SERS signal of CB[7] locating at 831 cm^-1^ is observed (Figure S14b, blue line), suggesting that the inclusive host-guest complex cannot situate in the hot spots region although the majority of MV^2+^ moieties are encapsulated by CB[7] in solution.


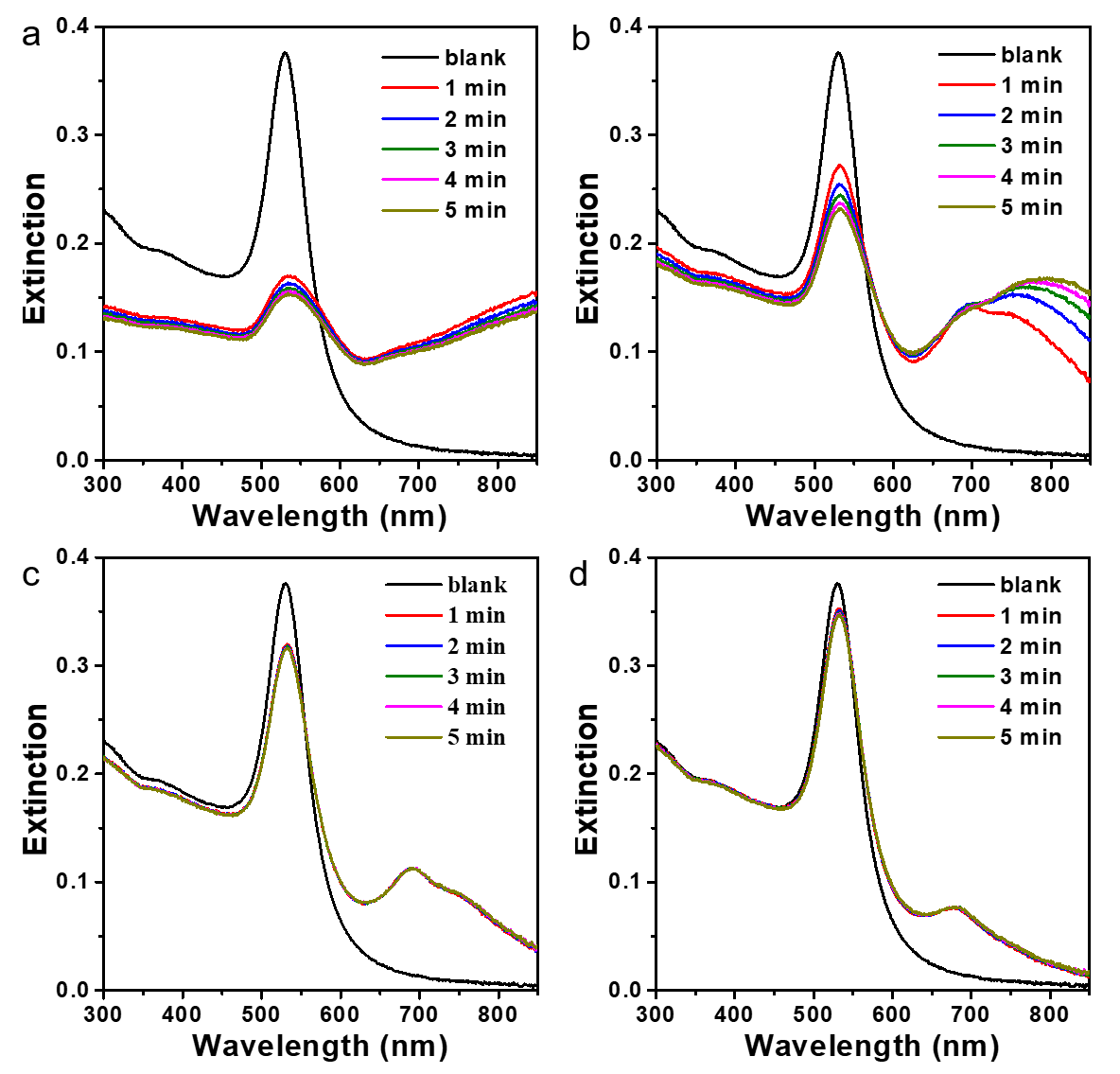


**Figure S16.** Time-dependent extinction spectra of the colloidal suspensions with different concentration of CB[7] with 2.5 μM MV^2+^2I**^−^**. The concentration of CB[7] were a) 0.31 μM, b) 0.62 μM, c) 2.5 μM, d) 5 μM and the spectra acquired at 1 min intervals for 5 min immediately after the mixing. The results demonstrate excess CB[7] reduced the aggregation of Au NPs.


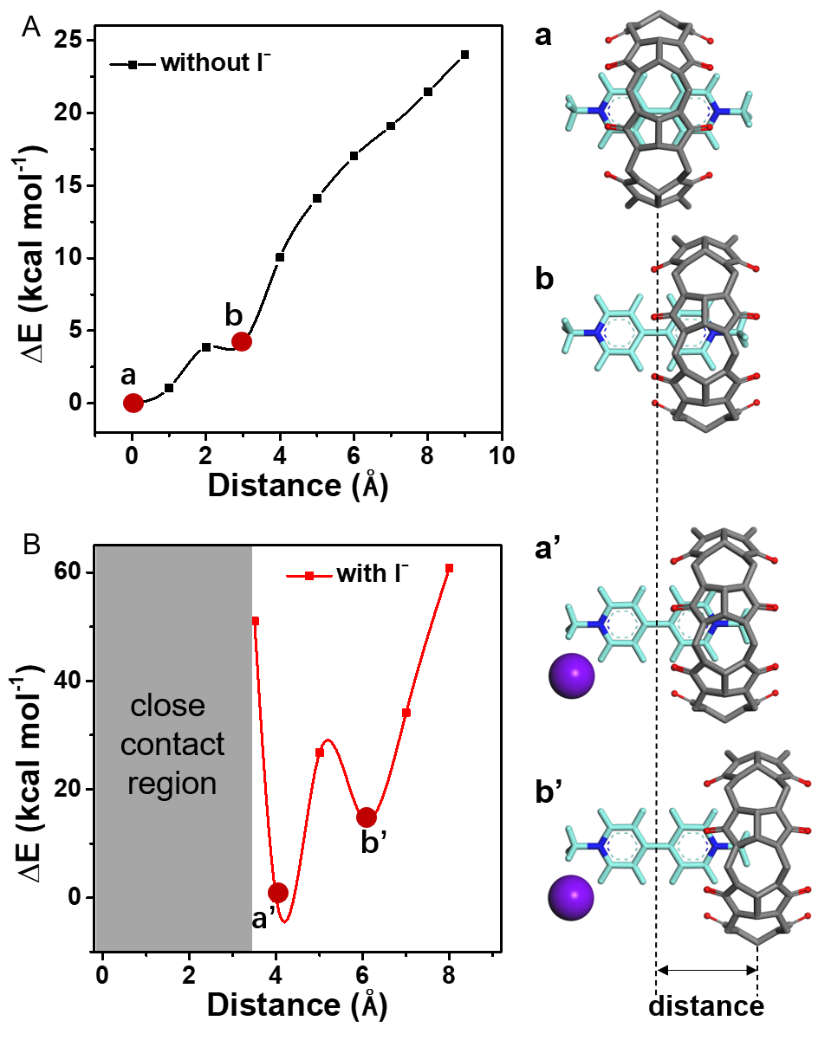


**Figure S17.** The relative total bonding energy (ΔΕ) profiles calculated for MV^2+^-CB[7] complex A) without or B) with I**^−^** as a function of distance. The total bonding energy of **a** or **a’** was set to zero respectively. All calculation work was performed at M06-2X/6-311G-LanL2DZ^4^ class on Gaussian 16^5^ with GD3^6^ empirical dispersion. The case with I^−^ shows a different profile compared with the case without I^−^, of which the two local minimums are both the exclusive complexation models. In other words, the significant electrostatic repulsion between I^−^ and carbonyl oxygens at CB[7] portal impedes the inclusive host-guest complexation.

**References**

1. Khan, M. S., Heger, D., Necas, M. & Sindelar, V. Remarkable salt effect on stability of supramolecular complex between modified cucurbit[6]uril and methylviologen in aqueous media. *J. Phys. Chem. B* **113**, 11054-11057 (2009).

2. Liu, S. et al. The cucurbit[n]uril family: prime components for self-sorting systems. *J. Am. Chem. Soc.* **127**, 15959-15967 (2005).

3. Descalzo, A. B. *et al*. The supramolecular chemistry of organic-inorganic hybrid materials. *Angew. Chem. Int. Ed.* **45**, 5924-5948 (2006).

4. Zhao, Y. & Truhlar, D. G. The M06 suite of density functionals for main group thermochemistry, thermochemical kinetics, noncovalent interactions, excited states, and transition elements: two new functionals and systematic testing of four M06-class functionals and 12 other functionals. *Theor. Chem. Acc.* **120**, 215-241 (2007).

5. Frisch, M. J. *et al*. Gaussian 16, Revision A.03; Gaussian, Inc., Wallingford CT, 2016.

6. Grimme, S., Antony, J., Ehrlich, S. & Krieg, H. A consistent and accurate ab initio parametrization of density functional dispersion correction (DFT-D) for the 94 elements H-Pu. *J. Chem. Phys.* **132**, 154104 (2010).
